# Distinct Licensing of IL-18 and IL-1β Secretion in Response to NLRP3 Inflammasome Activation

**DOI:** 10.1371/journal.pone.0045186

**Published:** 2012-09-18

**Authors:** Rebecca L. Schmidt, Laurel L. Lenz

**Affiliations:** 1 Integrated Department of Immunology, National Jewish Health, Denver, Colorado, United States of America; 2 Departments of Immunology and Microbiology, University of Colorado - Denver School of Medicine, Denver, Colorado, United States of America; University of California Merced, United States of America

## Abstract

Inflammasome activation permits processing of interleukins (IL)-1β and 18 and elicits cell death (pyroptosis). Whether these responses are independently licensed or are “hard-wired” consequences of caspase-1 (casp1) activity has not been clear. Here, we show that that each of these responses is independently regulated following activation of NLRP3 inflammasomes by a “non-canonical” stimulus, the secreted *Listeria monocytogenes* (Lm) p60 protein. Primed murine dendritic cells (DCs) responded to p60 stimulation with reactive oxygen species (ROS) production and secretion of IL-1β and IL-18 but not pyroptosis. Inhibitors of ROS production inhibited secretion of IL-1β, but did not impair IL-18 secretion. Furthermore, DCs from caspase-11 (casp11)-deficient 129S6 mice failed to secrete IL-1β in response to p60 but were fully responsive for IL-18 secretion. These findings reveal that there are distinct licensing requirements for processing of IL-18 versus IL-1β by NLRP3 inflammasomes.

## Introduction

Inflammasomes regulate the processing of pro-IL-1β and pro-IL-18 by caspase-1 (casp1) [1], as well as inflammatory cell death (pyroptosis) [2]. Inflammasome activation occurs in response to pathogen or damage-associated molecular patterns (PAMPs or DAMPs). In the case of NLRP3 inflammasomes, these factors include microbial proteins, crystalline urea, RNA, Alum, and ATP [3], [4], [5], [6], [7], [8]. The diversity of these activating stimuli implies that complex regulatory mechanisms govern NLRP3-dependent responses. Indeed, production of reactive oxygen species (ROS) and modification of the thioredoxin interacting protein, TXNIP, have been shown to cooperatively “license” NLRP3 inflammasomes to process IL-1β [9]. Recent findings further suggest that casp8 or casp11 can impact the response of NLRP3 inflammasomes to certain pathogen-derived “non-canonical” stimuli [10], [11]. It is not known whether ROS also participate in responses to such stimuli. Moreover, it remains unclear whether processing of IL-18 also requires ROS production or might instead be regulated by distinct ROS-independent licensing mechanisms.

Various members of the IL-1 cytokine family exert pro- or anti-inflammatory effects [12], [13]. Indeed, IL-18 and IL-1β act through distinct cell surface receptors and have distinct consequences during microbial infections [13], [14], [15], [16]. In some settings IL-18 can even counteract *in vivo* effects of IL-1β [12], [15], [17], [18]. Such findings suggest that tailoring the ratio of IL-1β versus IL-18 that is processed by inflammasomes might permit fine-tuning of inflammatory responses and influence infection outcomes. Yet, it is unknown whether activated NLRP3 inflammasomes can be differentially licensed to process IL-1β versus IL-18.

Lm is a bacterial pathogen that activates NLRP3 inflammasomes during infection [19], [20]. As a facultative intracellular pathogen, Lm can replicate both within the cytosol of host cells and extracellularly. The LLO hemolysin is required for Lm access and growth in the cytosol of many cultured mammalian cell types and for Lm virulence *in vivo* [21]. LLO also permits activation of NLRP3 inflammasomes in cultured cells [19], [22]. However, the specific Lm factors responsible for triggering NLRP3 inflammasome activation have previously remained undefined.

The *Listeria* protein p60 is abundantly secreted and essential for Lm virulence *in vivo* [23], [24], [25]. The p60 protein comprises an N-terminal portion with two LysM domains flanking an SH3 domain and a C-terminal portion with a repeat region and a NLPC/p60 domain [26]. The p60 NLPC/p60 domain shares homology with the *Bacillius subtilis* LytF endopeptidase and is predicted to mediate cleavage of peptide cross-links in bacterial peptidoglycan [23], [25], [27]. LysM domains are widely distributed in bacteria and plants and in several cases have been shown to bind carbohydrates in peptidoglycan or other glycoslyated biomolecules. [28], [29], [30], [31], [32], [33]. Bacterial SH3 domains similarly contribute to protein-protein or protein-glycan interactions [32], [33].

Recently, we showed that the Lm p60 protein acts on DCs to indirectly stimulate NK cell activation [27]. The activation of NK cells by p60 was an IL-18-dependent process, suggesting a role for inflammasome activation [23], [27]. Here, we mapped a region of the p60 protein that stimulates bone marrow-derived DCs (BMDCs) to secrete IL-18 and IL-1β by activating NLRP3 inflammasomes. Peptide derivatives of p60 elicited ROS production and stimulated NLRP3-dependent secretion of IL-1β and IL-18, but did not induce pyroptosis. In addition, we found that production and secretion of IL-1β, but not IL-18, required ROS production and did not occur in cells deficient for caspase-11. These data demonstrate that p60 is a non-canonical microbial activator of NLRP3 inflammasomes and reveal the existence of different licensing requirements for processing of IL-18 versus IL-1β.

## Materials and Methods

### Ethics Statement

This study was carried out in strict accordance with the recommendations of the Guide for the Care and Use of Laboratory Animals of the National Institutes of Health, the Public Health Service Policy on the Humane Care and Use of Laboratory Animals, and the Association for Assessment and Accreditation of Laboratory Animal Care. The protocols used were approved by the Institutional Animal Care and Use Committee at National Jewish Health (Protocol Permit AS2682-9-13). All efforts were made to minimize suffering.

### Mice

B6.*NLRP3^−/−^* mice originally from Dr. Richard Flavell (Yale University) were generously provided by Dr. Philippa Marrack (National Jewish Health). C57BL/6 mice (Jackson labs) and 129S6 mice (Taconic labs) were maintained in our colony. All mice were housed in the Biological Research Center of National Jewish Health.

### Bacterial Strains

Wild type *Listeria monocytogenes* 10403s and an isogenic strain with an in-frame deletion of p60 were used in these studies [25]. *Escherichia coli* TOP10 cells (Invitrogen) were used for expression of His-tagged proteins.

*Accession numbers* p60 (NCBI accession ZP_05235088.1).

### Production and Purification of Proteins and Synthetic Peptide

DNA coding for the mature p60, and the L1S, LysM2, or NLPC/p60 domain fragments were cloned into the pTrcHis-TOPO TA cloning vector (Invitrogen) as described previously [27]. IPTG-induced expression in *E.coli* was performed as described [27]. Polymyxin B columns were used as indicated by the manufacturer (Thermo Scientific) to remove LPS from purified proteins. In some experiments, purified proteins were boiled at 100°C for 10 minutes to denature protein structure or digested with Pronase protease mixture (Sigma-Aldrich) to ablate BMDC-stimulating activity. The LysM1 peptide (STVVVEAGDTLWGIAQSKGTTVDAIKKANNLTTDKIVPGQKLQVNNENAAE) was synthesized by Biomatick (Wilmington, DE).

### BMDC Culture and Stimulation

BMDCs were cultured and stimulated as described [27]. Cells were plated for >12 hours in antibiotic-free media, primed for 3 hours with 10 ng/ml ultra-pure LPS, 20 µg/ml polyinosine-polycytidylic acid (PIC) (Invivogen), 2 ng/ml recombinant mouse IL-12, or media alone. Purified p60 or L1S protein [30 µg/ml], synthetic LysM1 peptide [15 µg/ml], aluminum hydroxide (alum) (Accurate Chemical, Westbury, NY) [500 µg/ml], or 5 mM ATP (Invitrogen) were used as stimuli. For infection studies, the BMDC were infected with log phase 10403s wt or Δp60 Lm at MOI of 1, washed at 1 hpi and treated with 10 µg/ml gentamycin. For inhibitor studies, diphenyl iodonium chloride (DPI) [10 µM] (Sigma-Alderich,), Z-VAD-FMK, or Z-WEHD-FMK [10 µM] (Tocris, Minneapolis, MN) were applied 2 hours after TLR priming agents and 1 hour prior to protein stimulation.

### Western Blot, ELISA and LDH Assay

Lysates from BMDCs treated as above were probed with polyclonal α-IL-1β (R&D Systems) and monoclonal α-Caspase-11 Cas11.17D9 (Biolegend, San Diego, CA ). Concentrations of murine IL-1β and IL-18 were measured using commercial ELISA kits (BD Biosciences) on supernatants collected at the indicated times. LDH release was measured using the Cytotox96 cytotoxicity kit as per manufacturer instructions (Promega).

### QRT-PCR

Cell lysates were collected from BMDCs treated as above 6 hours post L1S stimulation. RNA was isolated using the RNeasy kit (Qiagen) and cDNA was obtained using the IM-Prom-II kit (Promega) according to the manufacturer’s instructions. Specific expression primers for IL-1β, IL-18, NLRP3, and GAPDH were synthesized as previously published [34], [35], [36], [37]. RT-PCR Primers are listed in Table 1. Quantitative PCR reactions using iTaq SYBR green supermix (BioRad) were performed and analyzed using the 7300 Real Time PCR instrument (AB Applied Biosystems). Data were computed using the 7300 ABS software (AB Applied Biosystems).

**Table 1:** Primers for Quantitative RT-PCR.

Gene	Sequence	Sequence
**GAPDH**	AACGACCCCTTCATTGAC	TCCACGACATACTCAGCAC
**IL-1β**	GCCCATCCTCTGTGACTCAT	AGGCCAC AGGTATTTTGTCG
**IL-18**	AAAGTGCCAGTGAACCC	TTTGATGTAAGTTAGTGAGAGTGA
**NLRP3**	TGCCTGTTCTTCCAGACTGGTGA	CACAGCACCCTCATGCCCGG

### ROS Production

BMDCs were washed in PBS and labeled with 10 µM of the ROS probe, CM-H_2_DCFDA (Invitrogen) for one hour. Washed cells were then treated with p60 protein ±20 µg/ml PIC or 2 ng/ml IL-12 for 4 hours. Live cells were washed in PBS and read at 485/528 nm. Fluorescence was assessed using a BioTek Synergy plate reader and expressed as arbitrary fluorescence units above background.

### Statistics

Statistic analysis was performed using Graph Pad Prism 5. *P* values were assessed using unpaired, two-tailed Student’s *t* tests (α = 0.05). In the figures, * denotes *P* values between 0.05 and 0.01, ** denotes *P* values between 0.01 and 0.001, and *** denotes *P* values < or  = 0.001. All error bars represent SEM. All experiments were performed in triplicate. Data shown are representative of at least three independent experiments.

## Results

### The Lm p60 Protein Induces IL-18 and IL-1β Secretion from BMDCs

**Figure 1:**
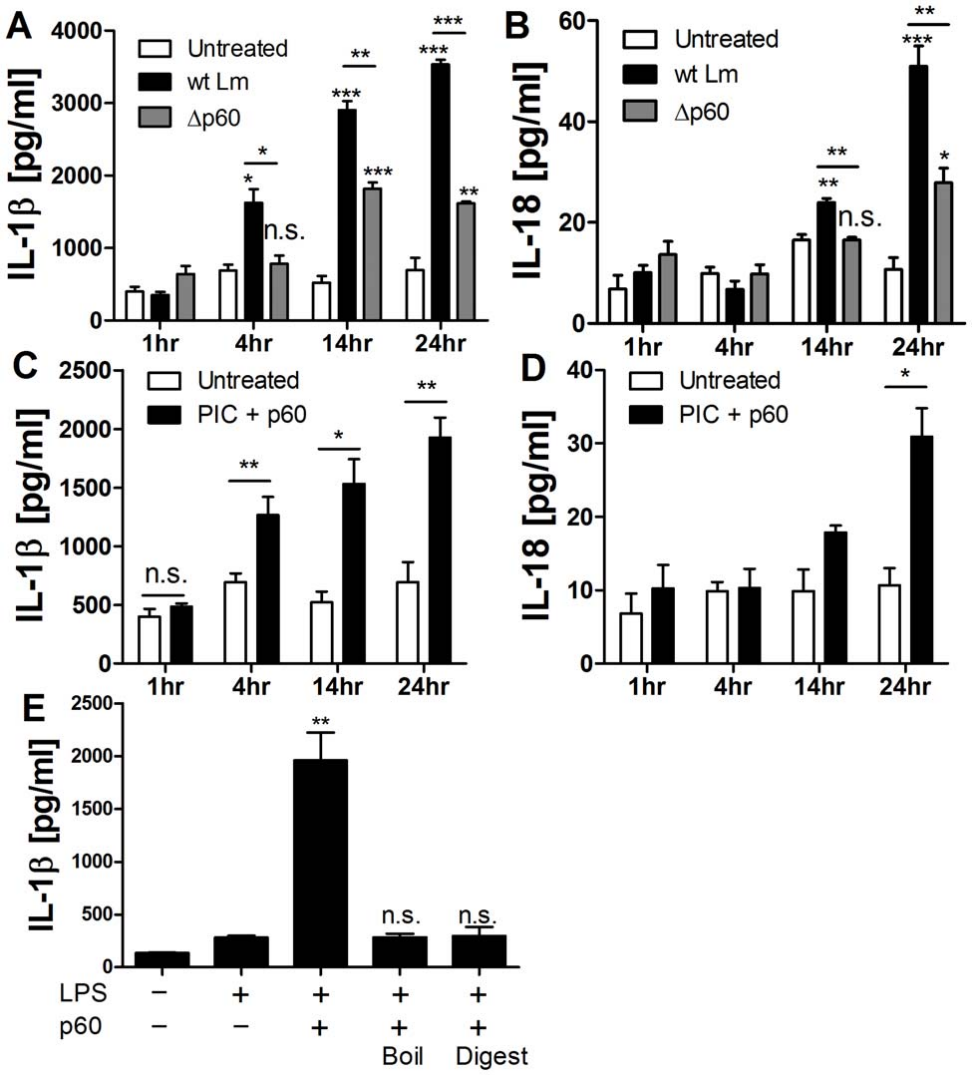
The Lm p60 protein induces IL-18 and IL-1β secretion from BMDCs. (**A, B**) BMDCs were infected with wt Lm or Δp60 mutant Lm for 1 h, (**C, D**) or primed with PIC then stimulated with purified p60 protein. Supernatants were collected at 1, 4, 14, and 24 h. Shown are concentrations of secreted IL-1 β and IL-18 as assessed by ELISA. (**E**) BMDCs were primed with LPS and then stimulated with purified p60, or p60 protein that had been boiled for 10 min or digested with protease treatment. Secreted IL-1β was measured by ELISA after 10 h. * denotes *P* values between 0.05 and 0.01, ** denotes *P* values between 0.01 and 0.001, and *** denotes *P* values < or  = 0.001. Error bars represent SEM. Experiments were performed in triplicate. Data shown are representative of at least three independent experiments.

Infection of BMDC by Lm induced secretion of IL-18 and IL-1β, which was significantly reduced upon infection with Δp60 Lm (Fig. 1A,B). Lm deficient for p60 are not growth deficient in BMDCs [27]. These results thus indicated that p60 directly or indirectly promotes processing of mature IL-1β and IL-18 during Lm-infection. To distinguish whether p60 acted directly on DCs or indirectly by altering the bacterial cell, we evaluated IL-1β and IL-18 secretion by BMDCs treated with detoxified recombinant p60 protein [27]. The treatment stimulated secretion of IL-18 and IL-1β, provided the BMDCs were first primed with either TLR agonists such as LPS and PIC, or inflammatory cytokines such as IL-12 (Fig. 1 C,D and see below). Consistent with the two-signal hypothesis for inflammasome activation [1], [38], treatment of the BMDC with TLR agonists alone failed to induce IL-1β or IL-18 secretion (Fig. 1C,D). The p60 protein failed to induce IL-1β secretion from BMDCs following denaturation by boiling or digestion by treatment with a protease cocktail (Fig. 1E), suggesting that additional *E.coli-*derived PAMPs did not contribute to IL-1β secretion in this system.

### Induction of IL-1β and IL-18 Secretion Maps to the p60 LysM1 Domain

**Figure 2:**
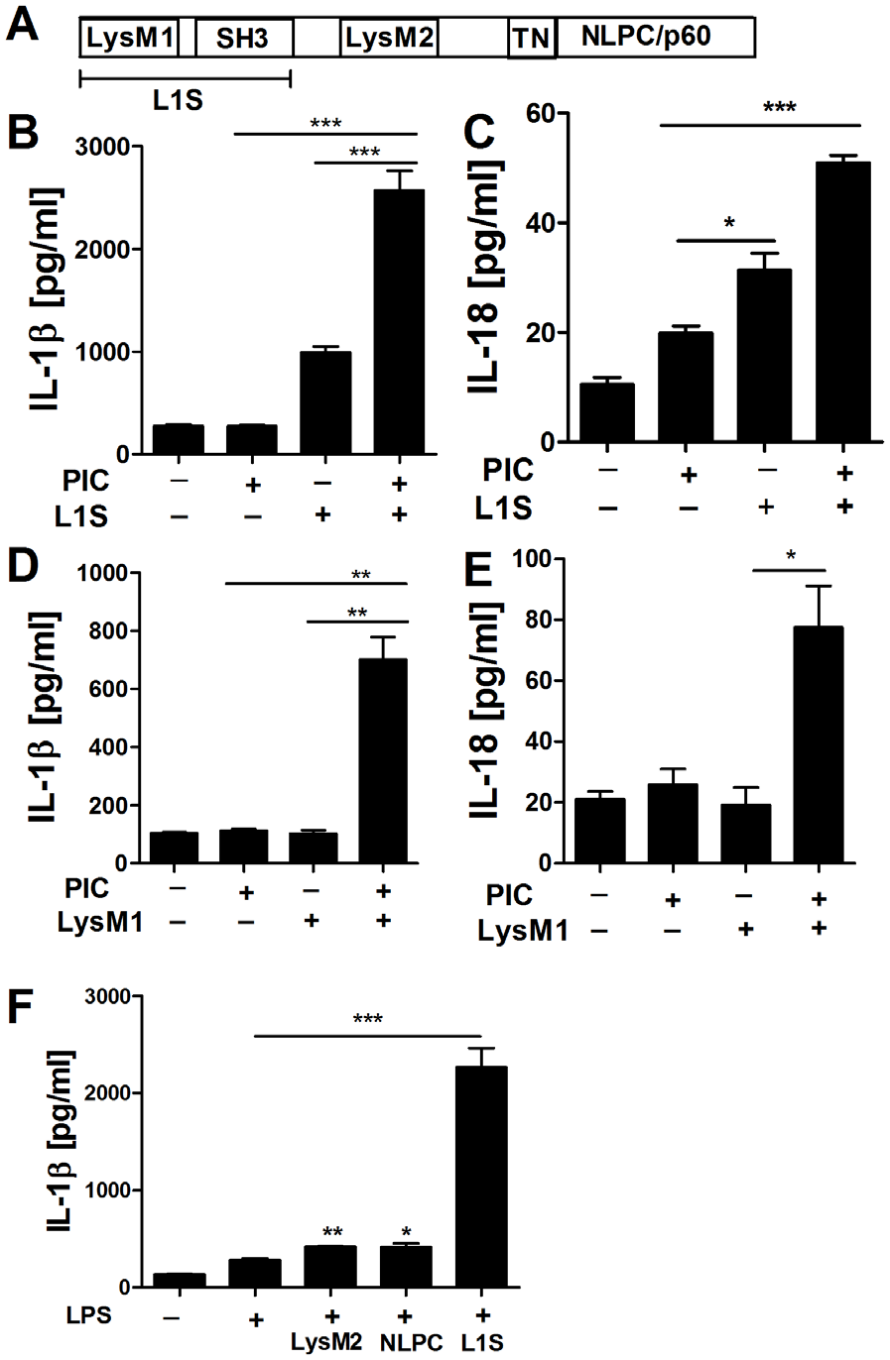
Induction of IL-1β and IL-18 secretion maps to the p60 LysM1 domain. (**A**) Domain map of p60. The protein contains two N-terminal LysM domains flanking a bacterial SH3 domain and a C-terminal NLPC/p60 catalytic domain. (**B, C**) BMDCs were primed with PIC then stimulated with purified L1S protein or (**D, E**) synthetic LysM1 peptide. (**F**) BMDCs were primed with LPS then stimulated with equimolar amounts of purified recombinant L1S, LysM2, or TN-NLPC/p60 protein fragments. Supernatants were collected at 10 h (IL-1β) or 24 h (IL-18) post-treatment to determine secreted cytokine concentrations by ELISA. * denotes *P* values between 0.05 and 0.01, ** denotes *P* values between 0.01 and 0.001, and *** denotes *P* values < or  = 0.001. Error bars represent SEM. Experiments were performed in triplicate. Data shown are representative of at least three independent experiments.

The Lm p60 protein contains a catalytic NLPC/p60 domain and two LysM domains which flank a bacterial SH3 domain (Fig. 2A). The ability of p60 to stimulate NK cell activation maps to a region comprising the LysM1 and SH3 domains, termed L1S [27]. The L1S region also induced IL-18 and IL-1β secretion when administered to BMDCs (Fig. 2B,C). Moreover, a synthetic peptide comprising the LysM1 domain sufficed to stimulate IL-1β and IL-18 secretion from PIC primed BMDCs (Fig.2D,E). Thus, post-translational modifications or bacterial contaminants were dispensable for the induction of IL-1β and IL-18 secretion by the LysM1 peptide. We additionally tested purified LysM2 and the NLPC/p60 domain fragments for BMDC-stimulating activity and found that in contrast to L1S, these peptides induced little IL-1β (Fig. 2F).

### Purified p60 Protein and L1S Polypeptide Activate the NLRP3 Inflammasome without Inducing Pyroptosis

**Figure 3:**
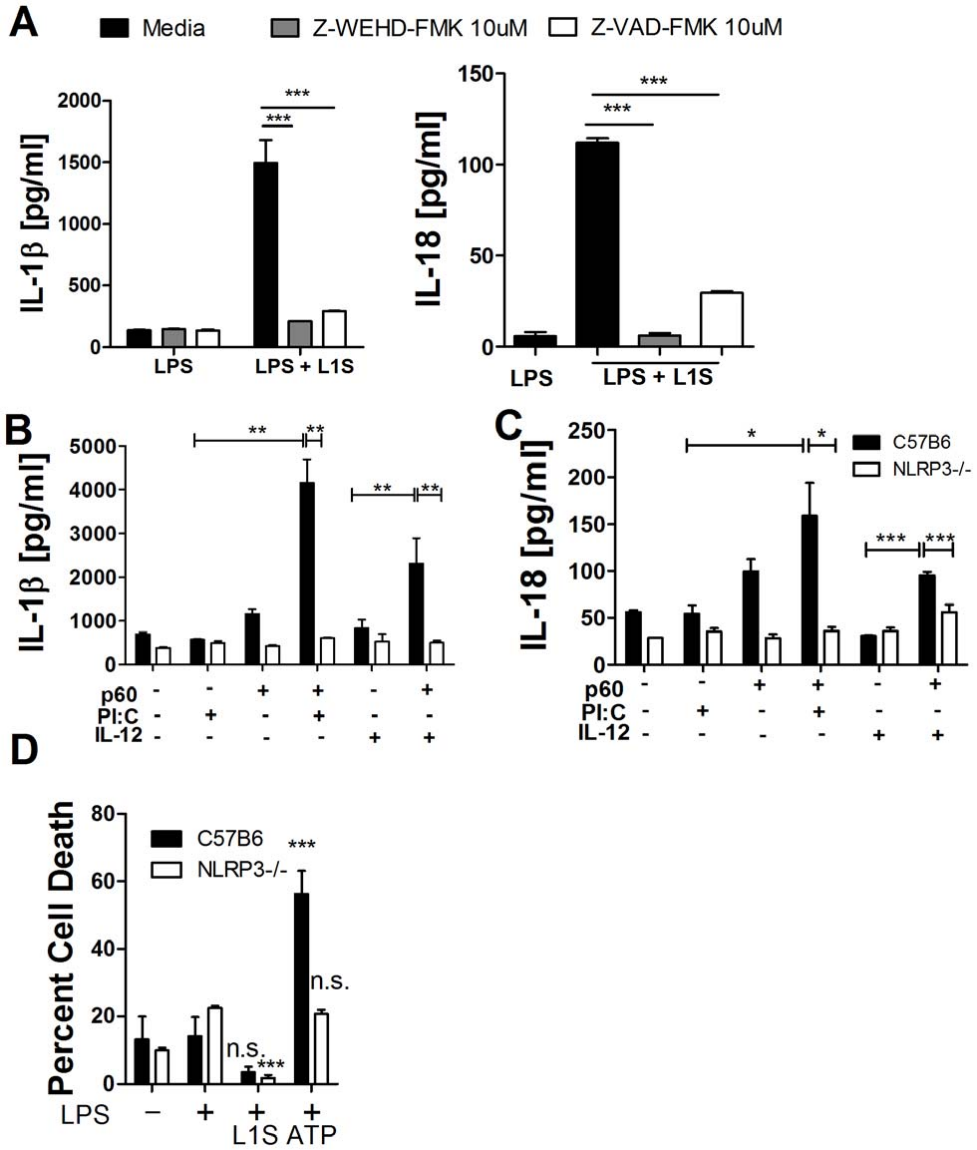
Purified p60 protein and L1S polypeptide activate the NLRP3 inflammasome without inducing pyroptosis. (**A**) BMDCs were primed with LPS, treated with caspase inhibitors Z-VAD-FMK [10 µM] or Z-WEHD-FMK [10 µM] for 1 h, and stimulated with purified L1S protein. Culture supernatants were analyzed by ELISA after 10 h (IL-1β) or 24 h (IL-18). (**B, C**) BMDCs from C57BL/6 or B6.*NLRP3^−/−^* mice were primed with PIC or IL-12 and then stimulated with purified p60 protein. Supernatants were collected at 10 h or 24 h post-treatment to measure concentrations of IL-1β and IL-18, respectively. (**D**) BMDC from C57BL/6 or B6.*NLRP3^−/−^* mice were primed with LPS and treated with L1S or ATP. Pyroptosis was assessed by LDH release after 8 h. * denotes *P* values between 0.05 and 0.01, ** denotes *P* values between 0.01 and 0.001, and *** denotes *P* values < or  = 0.001. Error bars represent SEM. Experiments were performed in triplicate. Data shown are representative of at least three independent experiments.

Casp 1-dependent and –independent proteolytic processing of IL-1 family cytokines has been reported [39], [40]. Hence, we asked whether specific inhibitors of casp-1, -4/11, and -5 (Z-WEHD-FMK) or a more generic caspase inhibitor (Z-VAD-FMK) abrogated Il-1β and IL-18 secretion in response to L1S peptide (Fig. 3A,B). Both inhibitors impaired IL-1β and IL-18 secretion by LPS-primed BMDCs, suggesting involvement of activated caspases. The requirement for priming in the response of BMDCs to p60 further evoked the requirement for priming to induce NLRP3 expression [38]. We thus asked whether BMDCs from B6.*NLRP3^−/−^* mice secreted IL-1β or IL-18 in response to treatment with p60 (Fig. 3B,C). The results indicated that NLRP3 was essential for secretion of both cytokines. Since activation of NLRP3 inflammasomes by extracellular adenosine triphosphate (ATP) and several other ligands is known to induce pyroptosis we also tested for lactose dehydrogenase (LDH) release (Fig. 3D). However, unlike ATP, the L1S peptide failed to induce cell death. Thus, the p60 LysM1 region induces activation of the NLRP3 inflammasome, but in a manner that fails to elicit the full spectrum of potential cellular responses.

### Distinct Requirements for ROS Production during IL-1β and IL-18 Secretion in Response to p60/L1S Stimulation

**Figure 4:**
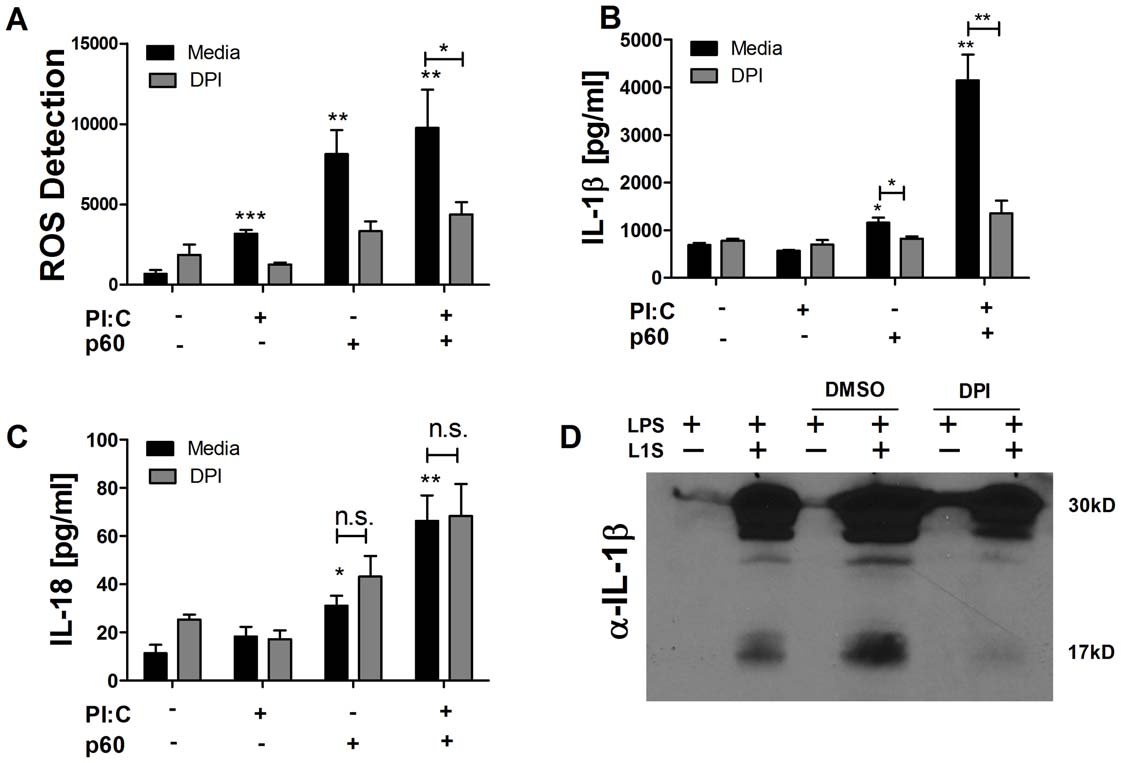
ROS are required for IL-1β but not IL-18 secretion in response to p60/L1S stimulation. (**A–C**)BMDC were primed where indicated with 20 µg/ml PIC, then stimulated where indicated with detoxified p60 protein. For DPI treatment, 10 µM DPI was added 2 h after priming but 1 h prior to p60 stimulation. (**A**) Cellular ROS levels were assessed 4 h post-treatment by measuring fluorescence of the ROS probe CM-H2DCFDA. (**B**) IL-1β and (**C**) IL-18 levels were measured by ELISA (**B**) 10 h or (**C**) 24 h post-treatment. (**D**) Cellular lysates were probed for IL-1β 10 h post-treatment with 10 ng/ml LPS with or without 30 ug/ml purified L1S. DMSO or DPI were added 2 h after priming but 1 h prior to L1S stimulation. * denotes *P* values between 0.05 and 0.01, ** denotes *P* values between 0.01 and 0.001, and *** denotes *P* values < or  = 0.001. Error bars represent SEM. Experiments were performed in triplicate. Data shown are representative of at least three independent experiments.

Like many activators of the NLRP3 inflammasome [3], [9], [41], the p60 protein triggered measurable ROS-dependent fluorescence of dichlorofluorescein (DCF) dye (Fig. 4A). Consistent with previous reports implicating ROS in regulation of NLRP3 inflammasome activation [3], [9], [41], the production of ROS in response to p60 was significantly greater than seen in response to PIC priming agent alone (Fig. 4A). This ROS production also correlated with secretion of IL-1β and IL-18 (Fig. 4B,C). Application of the inhibitor diphenyleneiodonium chloride (DPI) after priming, but prior to p60 stimulation, blocked ROS generation (Fig. 4A). These reductions in ROS correlated with reduced IL-1β secretion (Fig. 4B) and processing of pro-IL-1β (Fig. 4D). However, DPI treatment had no effect on IL-18 secretion (Fig. 4C, S1). Processing and secretion of IL-1β remained low at 24 hours during DPI treatment (Fig. S1), suggesting ROS inhibition did not simply delay IL-1β processing.

Recent reports revealed that DPI and other inhibitors of ROS production block the ability of priming with TLR agonists to induce expression of NLRP3 and pro forms of cytokines [12], [42], [43], [44]. To determine whether DPI treatment after addition of TLR agonists in our experiments above might have interfered with production of pro-IL-1β, pro-IL-18 and NLRP3, we examined RNA from cell lysates primed with LPS two hours prior to DPI treatment. BMDCs primed with 10 ng/ml LPS and stimulated 3 h later with 30 ug/ml L1S significantly up-regulated mRNA transcripts of NLRP3, pro-IL-1β, and pro-Il-18 (Fig. S2). DPI applied post-priming and 1 h before L1S stimulation did not inhibit such up-regulation (Fig. S2). These data confirm that inhibition of ROS production after TLR stimulation does not block NLRP3 expression [43], and suggest that ROS selectively stimulate NLRP3 inflammasome activity leading to IL-1β secretion. By contrast, IL-18 processing by the NLRP3 inflammasome does not require ROS.

### Lack of IL-1β but not IL-18 Secretion in Response to p60/L1S Stimulation of 129S6 Cells

**Figure 5:**
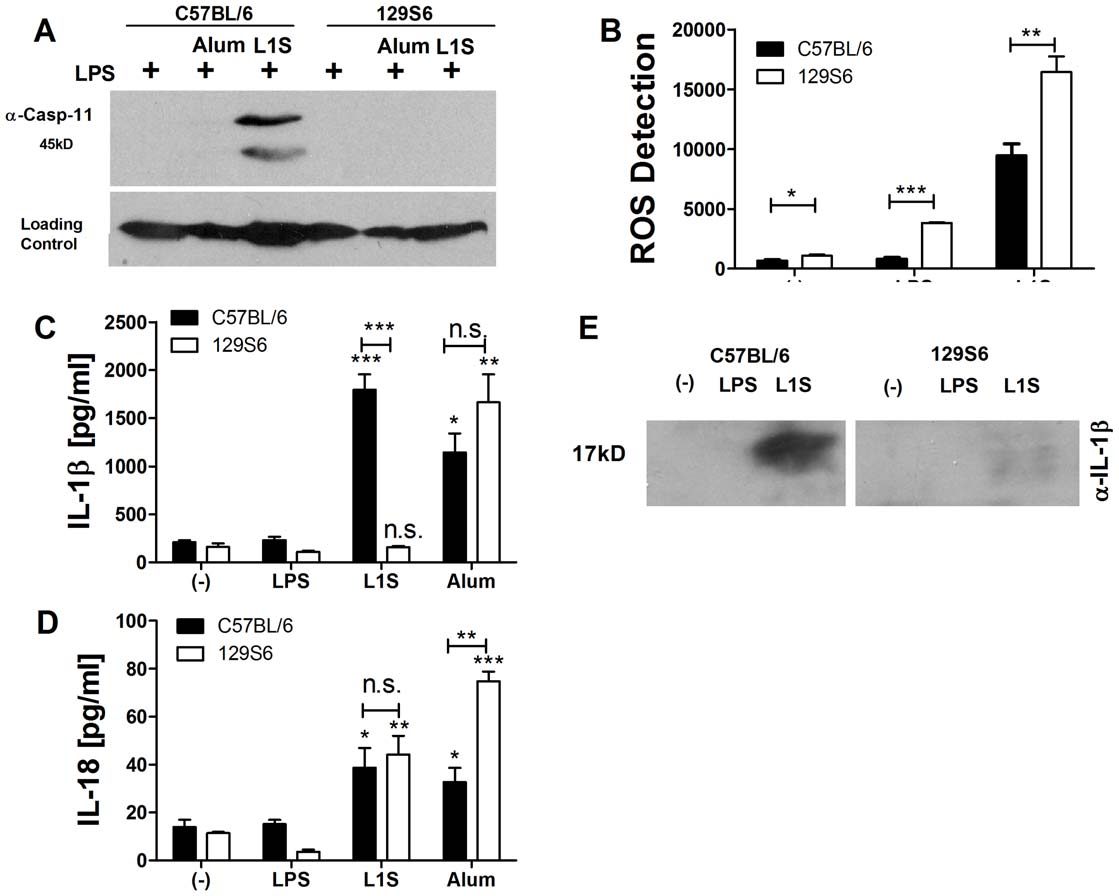
IL-1β and IL-18 secretion in response to p60/L1S stimulation differs in B6 and 129S6 mice. BMDC from C57BL/6 and 129S6 mice were primed with 10 ng/ml LPS and treated with detoxified p60/L1S or alum. (**A**) Cellular lysates from C57BL/6 and 129S6 BMDCs at 8 h post-treatment with L1S or alum were probed with anti-casp11. (**B**) Cellular ROS levels were assessed 4 h post-treatment by measuring fluorescence of the ROS probe CM-H2DCFDA. (**C**) IL-1β and (**D**) IL-18 concentrations were measured by ELISA (**C**) 10 h or (**D**) 24 h post-treatment. (**E**) Cellular lysates were probed for IL-1β at 10 h post-treatment with 10 ng/ml LPS with or without 30 ug/ml purified L1S. * denotes *P* values between 0.05 and 0.01, ** denotes *P* values between 0.01 and 0.001, and *** denotes *P* values < or  = 0.001. Error bars represent SEM. Experiments were performed in triplicate. Data shown are representative of at least three independent experiments.

NLRP3-dependent responses to certain “non-canonical” stimuli require casp11 [11]. Since 129S mice contain a null casp11 allele [11], we used BMDCs from these mice to ask if casp11 might affect p60/L1S-induced inflammasome activation. Activated casp11 was observed in LPS-primed BMDCs from C57BL/6 mice selectively when the cells were also stimulated with L1S (Fig. 5A). By contrast, we failed to observe substantial active casp11 in LPS-primed BMDCs alone or stimulated with the “canonical” NLRP3 agonist alum (Fig. 5A). Consistent with the findings of Kayagaki et al. (2011), casp11 was absent in lysates from 129S6 BMDCs (Fig. 5A). Given that casp11 activation was induced by L1S, we asked whether lack of casp11 in 129S6 BMDCs correlated with obviated ROS production and IL-1β or IL-18 secretion in response to L1S peptide. ROS production was not impaired in 129S6 mice stimulated with L1S (Fig. 5B). Likewise, BMDC stimulation with the canonical NLRP3 stimulus, alum, efficiently induced secretion of IL-1β in 129S6 BMDCs (Fig. 5C). However, in response to L1S processing and secretion of IL-1β was significantly (66–93%) reduced in the 129S6 BMDCs despite similar levels of pro-IL-1β and NLRP3 induction (Figs. 5C,D and S2). Conversely, secretion of IL-18 in response to alum or L1S treatments was not significantly altered in 129S6 BMDCs (Fig. 5E). These findings underscore the distinct requirements for processing of pro-IL1β and pro-IL18 in response to L1S stimulation.

Since there are numerous genetic differences between 129S6 and C57BL/6 mice, the observed differences in IL-1β and IL-18 secretion might conceivably be independent of casp11. We considered whether transcriptional regulation of the pro-cytokines or NLRP3 might be reduced in 129S6 BMDCs, but quantitative RT-PCR revealed similar induction of pro-IL-1β and NLRP3 after LPS/L1S stimulation (Fig. S2). Further, the levels of pro-IL-1β and NLRP3 induction were comparable between 129S6 and C57BL/6 BMDCs. Secretion of IL-18 in response to L1S treatment was not different in C57BL/6 and 129S6 BMDCs (Fig. 5E), and up-regulation of *il18* mRNA was not significantly reduced in the 129S6 BMDCs (Fig. S2H). However, basal *il18* was 3–20 fold higher in the 129S6 BMDCs (not shown). Overall, these findings are consistent with the hypothesis that the differential secretion of IL-1β in C57BL/6 and 129S6 BMDCs is not due to differential regulation of *nlrp3* or *il1b* expression, but rather to differential expression of casp11. Hence, the Lm p60 protein behaves as a “non-canonical” activator of the NLRP3 inflammasome.

## Discussion

Our findings have demonstrated a previously unappreciated plasticity in the cellular response to NLRP3 inflammasome activation. Using BMDCs from C57BL/6 and 129S6 mice we found that stimulation of cells with a “non-canonical” microbial activator of the NLRP3 inflammasome induced both IL-1β and IL-18 processing. Yet, secretion of IL-1β in response to this stimulus showed distinct requirements for ROS and casp11 when compared to IL-18. Thus, our findings demonstrate that there are different licensing requirements for NLRP3 inflammasome-dependent processing and secretion of IL-1β and IL-18.

ROS were previously shown to promote cleavage of pro-IL-1β by stimulating association of TXNIP with NLRP3 to “unlock” the ability of the NLRP3 inflammasome to activate casp1 and cleave pro-IL-1β [9]. The lack of requirements for ROS (and presumably casp11) in licensing of IL-18 secretion indicates that in the absence of ROS and/or casp11 host cells may selectively produce IL-18 in response to NLRP3 inflammasome activation. Such differential licensing may contribute to the selective production of IL-18, but not IL-1β, by epithelial cells during dextran induced colitis [45], [46]. However, our findings did not exclude the existence of additional unique requirements for licensing of pro-IL-18 secretion.

Recent studies reported that priming by TLR agonist stimulation promotes expression of NLRP3 as well as pro-IL-1β and pro-IL-18 [42], [43]. Treatment with ROS inhibitors such as DPI prior to TLR stimulation (DPI TLR) can block upregulation of these factors and thus impair inflammasome activation by preventing priming [43]. However, treatment with DPI after TLR priming (TLR DPI) did not impair inflammasome activation by the pore-forming activator nigericin [43]. In our experiments, DPI was added after 2 hours of TLR stimulation (TLR DPI). We further confirmed by our QRT-PCR data that this protocol for DPI treatment did not block induction of mRNA for NLRP3, pro-Il-1β, or pro-IL-18. Yet, we found that DPI inhibited secretion of IL-1β but not IL-18 by L1S-stimulated BMDCs. These results suggest that the mechanisms of L1S-mediated versus nigericin-mediated NLRP3 inflammasome activation are distinct. Namely, L1S-mediated inflammasome activation requires ROS at a post-priming stage, while nigericin-mediated activation does not.

Our results are consistent with a role for casp11 in L1S-mediated activation of the NLRP3 inflammasome, but proof of this interpretation would require experiments performed with caspase-11 deficient mice in a C57B/6 background. The lack of casp11 in the 129S6 strain is but one of several differences between this and C57BL/6. Indeed, we noted that basal *il18* transcript amounts were consistently higher in 129S6 mice (not shown). It is thus possible that increased availability of *il18* mRNA increases the amount of pro-IL-18 constitutively available for casp11-independent processing and secretion by 129S6 cells and contributes to their IL-18 secretion in response to L1S and alum treatments. The anthrax toxin-sensitive NLRP1b allele is also present in 129S6 but not C57BL/6 mice [47]. Further experiments would be needed to determine whether L1S might activate the NLRP1 inflammasome.

Although the Lm p60 protein activates NLRP3, expression of the Lm p60 protein is clearly important for bacterial pathogenicity during systemic infection [25]. How can this be the case given that NLRP3 expression is protective in many disease settings? IL-1β has been ascribed a pro-bacterial role in some bacterial infection models [15], and thus p60 might conceivably benefit Lm by inducing IL-1β. However, IL-1 and the IL-1 receptor, IL-1R1, reportedly promote host resistance to systemic Lm infection [12], [48], [49]. By contrast, IL-18 deficient mice were previously found to be resistant to systemic Lm infection [50]. The mechanistic basis for such pro-bacterial effects of IL-18 is unclear. However, IL-18 has antagonistic or regulatory effects on IL-1 during *Helicobacter* infection and in certain other models of inflammatory disease [14], [45], [46], [51]. Thus, we speculate that p60, and perhaps other protein activators of the NLRP3 inflammasome, may benefit bacterial and fungal pathogens by selectively triggering secretion of IL-18. Whether NLRP3 activation is beneficial or detrimental to a pathogen may depend on whether the context of such activation favors IL-18 versus IL-1β production. Moreover, NLRP3 activation may have no net effect in some settings. Indeed, while NLPR3 deficiency exacerbates host infection by *Mycobacterium marinum* it has little impact on susceptibility to *M. tuberculosis* [52]–[53].

In summary, our findings revealed that distinct co-factors license NLRP3 inflammasomes for induction of downstream responses. The ability to independently govern the processing and secretion of IL-1β, IL-18, and induction of pyroptosis likely evolved to permit fine-tuning of host cell responses to infectious and inflammatory stimuli. However, Lm and perhaps other pathogens may have co-evolved the ability to co-opt this differential regulation. Host molecules responsible for differential regulation of NLRP3 inflammasomes may be useful targets for anti-microbial and anti-inflammatory therapy.

## Supporting Information

Figure S1 **DPI inhibits IL-1B processing and secretion up to 24 hours post-treatment.** (**A–C**) BMDC were primed with PIC (where indicated), and then stimulated with p60 protein (where indicated). In DPI conditions, 10 µM DPI was added 2 hours after priming but 1 hour prior to p60 stimulation. IL-1β (**A**) and IL-18 (**B**) levels were measured by ELISA 24 hours post-treatment from the same experimental supernatants. (**C**) Cellular lysates were probed for IL-1β 24 hours post-treatment. * denotes *P* values between 0.05 and 0.01, ** denotes *P* values between 0.01 and 0.001 Error bars represent SEM. Experiments were performed in triplicate. Data shown represent two independent experiments.
(TIF)

Figure S2 **mRNA induction of IL-1β, NLRP3, and IL-18 after LPS/L1S treatment.** BMDC from C57B/6 or 129S6 mice were primed with 10 ng/ml LPS applied 3 hours prior to p60 stimulation. Where indicated, 10 µM DPI was added 2 hours after priming but 1 hour prior to p60 stimulation. Cell lysates were collected 6 hours post p60 stimulation. GAPDH levels (**A**, **E**) are shown relative to average C57B/6 untreated/unstimulated levels. mRNA fold induction of IL-1β (**B, F**), NLRP3 (**C, G**) and IL-18 (**D, H**), are shown relative to average unstimulated cells for each treatment condition or genotype. Error bars represent SEM. Experiments were performed in triplicate. Data shown represent three independent experiments.
(TIF)

## References

[1] Schroder K, Tschopp J (2010) The inflammasomes. Cell 140: 821–832. 20303873 10.1016/j.cell.2010.01.040

[2] Bergsbaken T, Fink SL, Cookson BT (2009) Pyroptosis: host cell death and inflammation. Nat Rev Microbiol 7: 99–109. 19148178 10.1038/nrmicro2070 PMC2910423

[3] Allen IC, Scull MA, Moore CB, Holl EK, McElvania-TeKippe E, et al (2009) The NLRP3 inflammasome mediates in vivo innate immunity to influenza A virus through recognition of viral RNA. Immunity 30: 556–565. 19362020 10.1016/j.immuni.2009.02.005 PMC2803103

[4] Gross O, Poeck H, Bscheider M, Dostert C, Hannesschlager N, et al (2009) Syk kinase signalling couples to the Nlrp3 inflammasome for anti-fungal host defence. Nature 459: 433. 19339971 10.1038/nature07965

[5] Ichinohe T, Pang IK, Iwasaki A (2010) Influenza virus activates inflammasomes via its intracellular M2 ion channel. Nat Immunol 11: 404–410. 20383149 10.1038/ni.1861 PMC2857582

[6] Ritter M, Gross O, Kays S, Ruland J, Nimmerjahn F, et al (2010) Schistosoma mansoni triggers Dectin-2, which activates the Nlrp3 inflammasome and alters adaptive immune responses. Proc Natl Acad Sci U S A 107: 20459–20464. 21059925 10.1073/pnas.1010337107 PMC2996650

[7] Eisenbarth SC, Colegio OR, O'Connor W, Sutterwala FS, Flavell RA (2008) Crucial role for the Nalp3 inflammasome in the immunostimulatory properties of aluminium adjuvants. Nature 453: 1122–1126. 18496530 10.1038/nature06939 PMC4804622

[8] Gattorno M, Tassi S, Carta S, Delfino L, Ferlito F, et al (2007) Pattern of interleukin-1beta secretion in response to lipopolysaccharide and ATP before and after interleukin-1 blockade in patients with CIAS1 mutations. Arthritis Rheum 56: 3138–3148. 17763411 10.1002/art.22842

[9] Zhou R, Tardivel A, Thorens B, Choi I, Tschopp J (2010) Thioredoxin-interacting protein links oxidative stress to inflammasome activation. Nat Immunol 11: 136–140. 20023662 10.1038/ni.1831

[10] Gringhuis SI, Kaptein TM, Wevers BA, Theelen B, van der Vlist M, et al (2012) Dectin-1 is an extracellular pathogen sensor for the induction and processing of IL-1beta via a noncanonical caspase-8 inflammasome. Nat Immunol 13: 246–254. 22267217 10.1038/ni.2222

[11] Kayagaki N, Warming S, Lamkanfi M, Vande Walle L, Louie S, et al (2011) Non-canonical inflammasome activation targets caspase-11. Nature 479: 117–121. 22002608 10.1038/nature10558

[12] Dinarello CA (2009) Immunological and inflammatory functions of the interleukin-1 family. Annu Rev Immunol 27: 519–550. 19302047 10.1146/annurev.immunol.021908.132612

[13] Sahoo M, Ceballos-Olvera I, del Barrio L, Re F (2011) Role of the inflammasome, IL-1beta, and IL-18 in bacterial infections. ScientificWorldJournal 11: 2037–2050. 22125454 10.1100/2011/212680 PMC3217589

[14] Hitzler I, Sayi A, Kohler E, Engler DB, Koch KN, et al.. (2012) Caspase-1 Has Both Pro-Inflammatory and Regulatory Properties in Helicobacter Infections, Which Are Differentially Mediated by Its Substrates IL-1beta and IL-18. J Immunol. 10.4049/jimmunol.1103212 22403439

[15] Ceballos-Olvera I, Sahoo M, Miller MA, Del Barrio L, Re F (2011) Inflammasome-dependent pyroptosis and IL-18 protect against Burkholderia pseudomallei lung infection while IL-1beta is deleterious. PLoS Pathog 7: e1002452. 22241982 10.1371/journal.ppat.1002452 PMC3248555

[16] Mariathasan S, Newton K, Monack DM, Vucic D, French DM, et al (2004) Differential activation of the inflammasome by caspase-1 adaptors ASC and Ipaf. Nature 430: 213–218. 15190255 10.1038/nature02664

[17] Nakanishi K, Yoshimoto T, Tsutsui H, Okamura H (2001) Interleukin-18 regulates both Th1 and Th2 responses. Annu Rev Immunol 19: 423–474. 11244043 10.1146/annurev.immunol.19.1.423

[18] Biet F, Locht C, Kremer L (2002) Immunoregulatory functions of interleukin 18 and its role in defense against bacterial pathogens. J Mol Med 80: 147–162. 11894141 10.1007/s00109-001-0307-1

[19] Mariathasan S, Weiss DS, Newton K, McBride J, O'Rourke K, et al (2006) Cryopyrin activates the inflammasome in response to toxins and ATP. Nature 440: 228–232. 16407890 10.1038/nature04515

[20] Warren SE, Mao DP, Rodriguez AE, Miao EA, Aderem A (2008) Multiple Nod-like receptors activate caspase 1 during Listeria monocytogenes infection. J Immunol 180: 7558–7564. 18490757 10.4049/jimmunol.180.11.7558 PMC2991040

[21] Camejo A, Carvalho F, Reis O, Leitao E, Sousa S, et al (2011) The arsenal of virulence factors deployed by Listeria monocytogenes to promote its cell infection cycle. Virulence 2: 379–394. 21921683 10.4161/viru.2.5.17703

[22] Meixenberger K, Pache F, Eitel J, Schmeck B, Hippenstiel S, et al (2010) Listeria monocytogenes-infected human peripheral blood mononuclear cells produce IL-1beta, depending on listeriolysin O and NLRP3. J Immunol 184: 922–930. 20008285 10.4049/jimmunol.0901346

[23] Humann J, Bjordahl R, Andreasen K, Lenz LL (2007) Expression of the p60 autolysin enhances NK cell activation and is required for listeria monocytogenes expansion in IFN-gamma-responsive mice. J Immunol 178: 2407–2414. 17277147 10.4049/jimmunol.178.4.2407

[24] Lenz LL, Portnoy DA (2002) Identification of a second Listeria secA gene associated with protein secretion and the rough phenotype. Mol Microbiol 45: 1043–1056. 12180923 10.1046/j.1365-2958.2002.03072.x

[25] Lenz LL, Mohammadi S, Geissler A, Portnoy DA (2003) SecA2-dependent secretion of autolytic enzymes promotes Listeria monocytogenes pathogenesis. Proc Natl Acad Sci U S A 100: 12432–12437. 14527997 10.1073/pnas.2133653100 PMC218775

[26] Wuenscher MD, Kohler S, Bubert A, Gerike U, Goebel W (1993) The iap gene of Listeria monocytogenes is essential for cell viability, and its gene product, p60, has bacteriolytic activity. J Bacteriol 175: 3491–3501. 8099071 10.1128/jb.175.11.3491-3501.1993 PMC204749

[27] Schmidt RL, Filak HC, Lemon JD, Potter TA, Lenz LL (2011) A LysM and SH3-domain containing region of the Listeria monocytogenes p60 protein stimulates accessory cells to promote activation of host NK cells. PLoS Pathog 7: e1002368. 22072975 10.1371/journal.ppat.1002368 PMC3207947

[28] de Jonge R, Thomma BPHJ (2009) Fungal LysM effectors: extinguishers of host immunity? Trends in microbiology 17: 151. 19299132 10.1016/j.tim.2009.01.002

[29] Gust AA, Willmann R, Desaki Y, Grabherr HM, Nürnberger T (2012) Plant LysM proteins: modules mediating symbiosis and immunity. Trends in plant science 17: 495. 22578284 10.1016/j.tplants.2012.04.003

[30] Bateman A, Bycroft M (2000) The structure of a LysM domain from E. coli membrane-bound lytic murein transglycosylase D (MltD). J Mol Biol 299: 1113–1119. 10843862 10.1006/jmbi.2000.3778

[31] Buist G, Steen A, Kok J, Kuipers OP (2008) LysM, a widely distributed protein motif for binding to (peptido)glycans. Mol Microbiol 68: 838–847. 18430080 10.1111/j.1365-2958.2008.06211.x

[32] Leon E, Navarro-Aviles G, Santiveri CM, Flores-Flores C, Rico M, et al (2010) A bacterial antirepressor with SH3 domain topology mimics operator DNA in sequestering the repressor DNA recognition helix. Nucleic Acids Res 38: 5226–5241. 20410074 10.1093/nar/gkq277 PMC2926617

[33] Morton CJ, Campbell ID (1994) SH3 domains. Molecular ‘Velcro’. Curr Biol 4: 615–617. 7953536 10.1016/s0960-9822(00)00134-2

[34] Dai J-N, Zong Y, Zhong L-M, Li Y-M, Zhang W, et al (2011) Gastrodin Inhibits Expression of Inducible NO Synthase, Cyclooxygenase-2 and Proinflammatory Cytokines in Cultured LPS-Stimulated Microglia via MAPK Pathways. PLoS ONE 6: e21891. 21765922 10.1371/journal.pone.0021891 PMC3134470

[35] Jeudy S, Wardrop KE, Alessi A, Dominov JA (2011) Bcl-2 Inhibits the Innate Immune Response during Early Pathogenesis of Murine Congenital Muscular Dystrophy. PLoS ONE 6: e22369. 21850221 10.1371/journal.pone.0022369 PMC3151242

[36] Sun H, Lu J, Xu X, Jin S, Wang X, et al (2005) Histone acetyltransferase activity of p300 enhances the activation of IL-18 promoter. Journal of Cellular Biochemistry 94: 566. 15543578 10.1002/jcb.20194

[37] Pai RK, Pennini ME, Tobian AA, Canaday DH, Boom WH, et al (2004) Prolonged toll-like receptor signaling by Mycobacterium tuberculosis and its 19-kilodalton lipoprotein inhibits gamma interferon-induced regulation of selected genes in macrophages. Infect Immun 72: 6603–6614. 15501793 10.1128/IAI.72.11.6603-6614.2004 PMC523004

[38] Latz E (2010) The inflammasomes: mechanisms of activation and function. Curr Opin Immunol 22: 28–33. 20060699 10.1016/j.coi.2009.12.004 PMC2844336

[39] Mayer-Barber KD, Barber DL, Shenderov K, White SD, Wilson MS, et al (2010) Caspase-1 independent IL-1beta production is critical for host resistance to mycobacterium tuberculosis and does not require TLR signaling in vivo. J Immunol 184: 3326–3330. 20200276 10.4049/jimmunol.0904189 PMC3420351

[40] Provoost S, Maes T, Pauwels NS, Vanden Berghe T, Vandenabeele P, et al (2011) NLRP3/caspase-1-independent IL-1beta production mediates diesel exhaust particle-induced pulmonary inflammation. J Immunol 187: 3331–3337. 21844393 10.4049/jimmunol.1004062

[41] Said-Sadier N, Padilla E, Langsley G, Ojcius DM (2010) Aspergillus fumigatus stimulates the NLRP3 inflammasome through a pathway requiring ROS production and the Syk tyrosine kinase. PLoS One 5: e10008. 20368800 10.1371/journal.pone.0010008 PMC2848854

[42] Bauernfeind FG, Horvath G, Stutz A, Alnemri ES, MacDonald K, et al (2009) Cutting edge: NF-kappaB activating pattern recognition and cytokine receptors license NLRP3 inflammasome activation by regulating NLRP3 expression. J Immunol 183: 787–791. 19570822 10.4049/jimmunol.0901363 PMC2824855

[43] Bauernfeind F, Bartok E, Rieger A, Franchi L, Nunez G, et al (2011) Cutting edge: reactive oxygen species inhibitors block priming, but not activation, of the NLRP3 inflammasome. J Immunol 187: 613–617. 21677136 10.4049/jimmunol.1100613 PMC3131480

[44] Bulua AC, Simon A, Maddipati R, Pelletier M, Park H, et al (2011) Mitochondrial reactive oxygen species promote production of proinflammatory cytokines and are elevated in TNFR1-associated periodic syndrome (TRAPS). J Exp Med 208: 519–533. 21282379 10.1084/jem.20102049 PMC3058571

[45] Dupaul-Chicoine J, Yeretssian G, Doiron K, Bergstrom KS, McIntire CR, et al (2010) Control of intestinal homeostasis, colitis, and colitis-associated colorectal cancer by the inflammatory caspases. Immunity 32: 367–378. 20226691 10.1016/j.immuni.2010.02.012

[46] Zaki MH, Boyd KL, Vogel P, Kastan MB, Lamkanfi M, et al (2010) The NLRP3 inflammasome protects against loss of epithelial integrity and mortality during experimental colitis. Immunity 32: 379–391. 20303296 10.1016/j.immuni.2010.03.003 PMC2982187

[47] Boyden ED, Dietrich WF (2006) Nalp1b controls mouse macrophage susceptibility to anthrax lethal toxin. Nat Genet 38: 240–244. 16429160 10.1038/ng1724

[48] Labow M, Shuster D, Zetterstrom M, Nunes P, Terry R, et al (1997) Absence of IL-1 signaling and reduced inflammatory response in IL-1 type I receptor-deficient mice. J Immunol 159: 2452–2461. 9278338

[49] Hirsch E, Irikura VM, Paul SM, Hirsh D (1996) Functions of interleukin 1 receptor antagonist in gene knockout and overproducing mice. Proc Natl Acad Sci U S A 93: 11008–11013. 8855299 10.1073/pnas.93.20.11008 PMC38274

[50] Lochner M, Kastenmuller K, Neuenhahn M, Weighardt H, Busch DH, et al (2008) Decreased susceptibility of mice to infection with Listeria monocytogenes in the absence of interleukin-18. Infect Immun 76: 3881–3890. 18573894 10.1128/IAI.01651-07 PMC2519415

[51] Andoh T, Kishi H, Motoki K, Nakanishi K, Kuraishi Y, et al (2008) Protective effect of IL-18 on kainate- and IL-1 beta-induced cerebellar ataxia in mice. J Immunol 180: 2322–2328. 18250441 10.4049/jimmunol.180.4.2322

[52] Carlsson F, Kim J, Dumitru C, Barck KH, Carano RA, et al (2010) Host-detrimental role of Esx-1-mediated inflammasome activation in mycobacterial infection. PLoS Pathog 6: e1000895. 20463815 10.1371/journal.ppat.1000895 PMC2865529

[53] McElvania Tekippe E, Allen IC, Hulseberg PD, Sullivan JT, McCann JR, et al (2010) Granuloma formation and host defense in chronic Mycobacterium tuberculosis infection requires PYCARD/ASC but not NLRP3 or caspase-1. PLoS One 5: e12320. 20808838 10.1371/journal.pone.0012320 PMC2924896

