# Supplementary figures and images for: Distinct Licensing of IL-18 and IL-1β Secretion in Response to NLRP3 Inflammasome Activation
Source: PLoS One. 2012 Sep 18;7(9):e45186. doi: 10.1371/journal.pone.0045186 (PMC3445464; doi:10.1371/journal.pone.0045186)

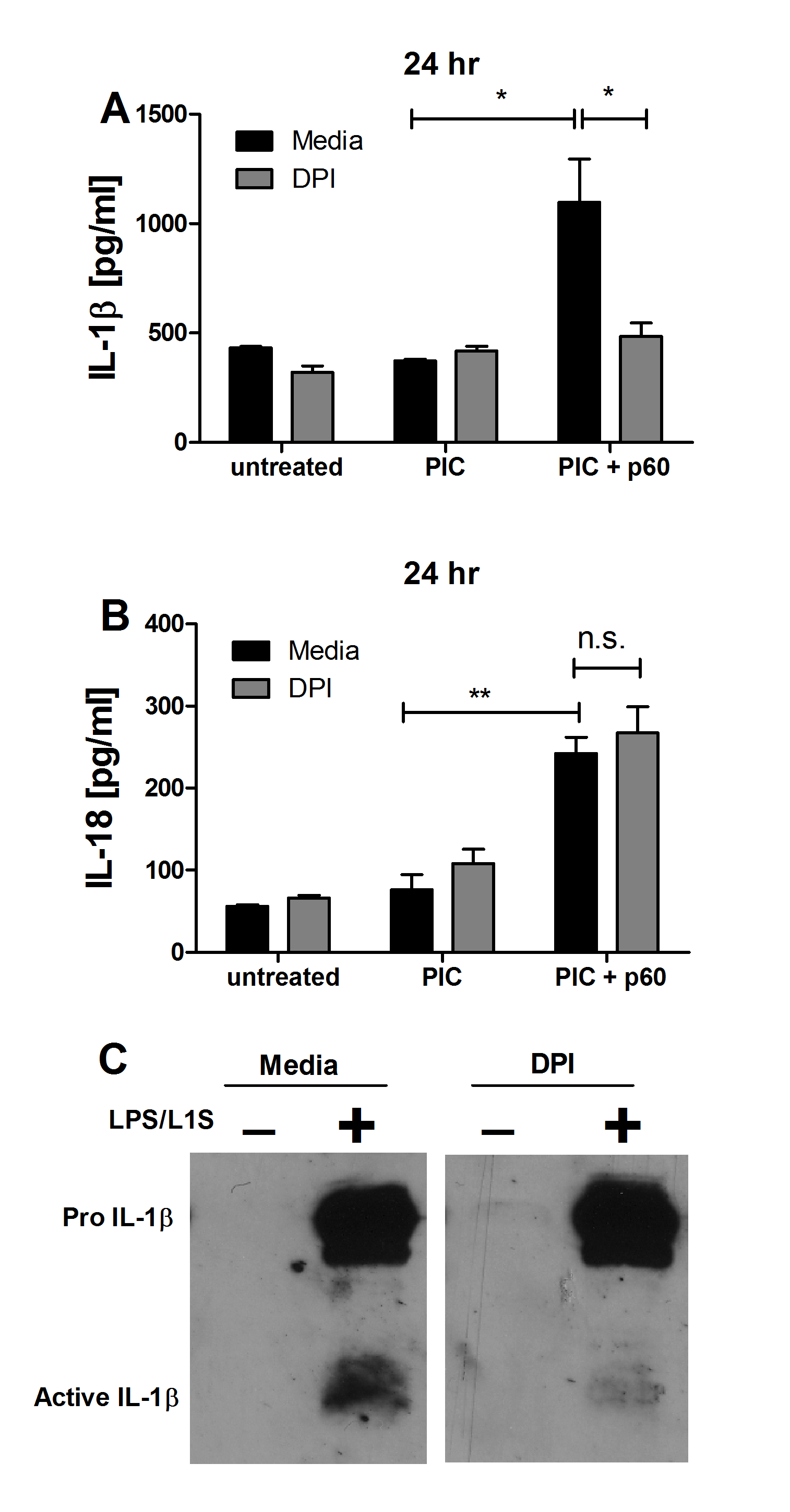

Supplement: Figure S1 — DPI inhibits IL-1B processing and secretion up to 24 hours post-treatment. (A–C) BMDC were primed with PIC (where indicated), and then stimulated with p60 protein (where indicated). In DPI conditions, 10 µM DPI was added 2 hours after priming but 1 hour prior to p60 stimulation. IL-1β (A) and IL-18 (B) levels were measured by ELISA 24 hours post-treatment from the same experimental supernatants. (C) Cellular lysates were probed for IL-1β 24 hours post-treatment. * denotes P values between 0.05 and 0.01, ** denotes P values between 0.01 and 0.001 Error bars represent SEM. Experiments were performed in triplicate. Data shown represent two independent experiments. (TIF) [file pone.0045186.s001.tif]

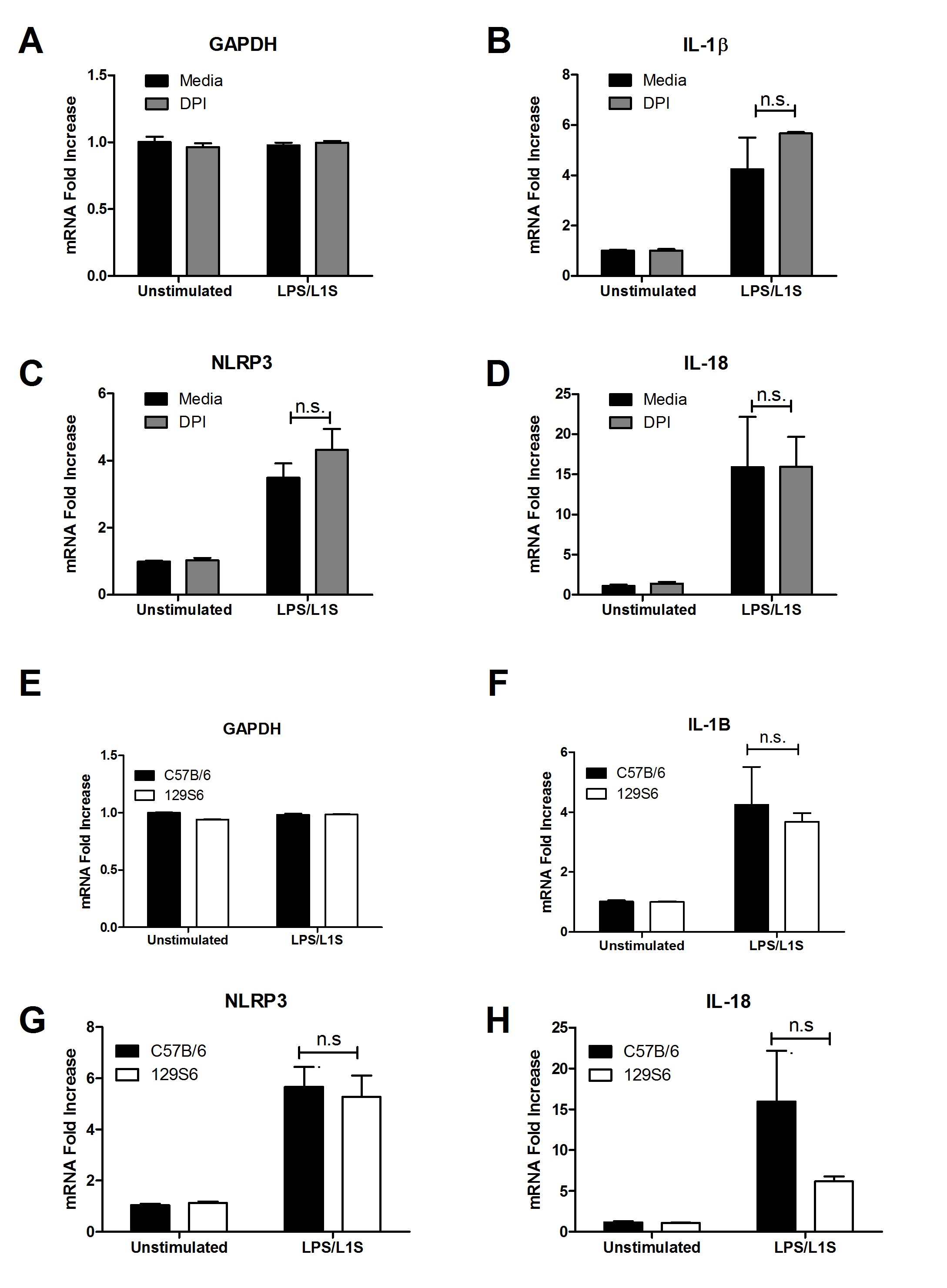

Supplement: Figure S2 — mRNA induction of IL-1β, NLRP3, and IL-18 after LPS/L1S treatment. BMDC from C57B/6 or 129S6 mice were primed with 10 ng/ml LPS applied 3 hours prior to p60 stimulation. Where indicated, 10 µM DPI was added 2 hours after priming but 1 hour prior to p60 stimulation. Cell lysates were collected 6 hours post p60 stimulation. GAPDH levels (A, E) are shown relative to average C57B/6 untreated/unstimulated levels. mRNA fold induction of IL-1β (B, F), NLRP3 (C, G) and IL-18 (D, H), are shown relative to average unstimulated cells for each treatment condition or genotype. Error bars represent SEM. Experiments were performed in triplicate. Data shown represent three independent experiments. (TIF) [file pone.0045186.s002.tif]
